# Pupil Dilation Dynamics Track Attention to High-Level Information

**DOI:** 10.1371/journal.pone.0102463

**Published:** 2014-08-27

**Authors:** Olivia E. Kang, Katherine E. Huffer, Thalia P. Wheatley

**Affiliations:** Psychological & Brain Sciences, Dartmouth College, Hanover, NH, United States of America; Davis, United States of America

## Abstract

It has long been thought that the eyes index the inner workings of the mind. Consistent with this intuition, empirical research has demonstrated that pupils dilate as a consequence of attentional effort. Recently, Smallwood et al. (2011) demonstrated that pupil dilations not only provide an index of overall attentional effort, but are time-locked to stimulus changes during attention (but not during mind-wandering). This finding suggests that pupil dilations afford a dynamic readout of conscious information processing. However, because stimulus onsets in their study involved shifts in luminance as well as information, they could not determine whether this coupling of stimulus and pupillary dynamics reflected attention to low-level (luminance) or high-level (information) changes. Here, we replicated the methodology and findings of Smallwood et al. (2011) while controlling for luminance changes. When presented with isoluminant digit sequences, participants' pupillary dilations were synchronized with stimulus onsets when attending, but not when mind-wandering. This replicates Smallwood et al. (2011) and clarifies their finding by demonstrating that stimulus-pupil coupling reflects online cognitive processing beyond sensory gain.

## Introduction

Poets, philosophers, and artists throughout history have considered eyes to be portals to the mind. Science supports this intuition: eyes broadcast mind and animacy better than any other facial feature [1], with specific ocular cues linked to emotion (scleral size, [2]), information processing (eye-blinks, [3]), and intention (gaze direction, [4]). The pupil, especially, has been investigated as an index of mental states, given its tendency to dilate to salient stimuli such as emotionally arousing pictures [5], painful stimulation [6], and task-relevant numbers [7].

Pupil dilations due to changes in informational salience are much smaller than pupil dilations due to changes in luminance [8]. This difference in size reflects different biological pathways. Information-related dilations are associated with sympathetic activation of the superior sympathetic ganglion, and rarely exceed .5 mm [8], [9]. In contrast, luminance-related dilations index parasympathetic activation of the Edinger-Westphal nucleus [10] and are generally between 2–4 mm [8], [11]. For this reason, pupil dilations that index informational salience are only reliably measured under conditions of controlled light [7], [12]–[14].

Although previous studies have been careful to control luminance to reveal salience-related dilations, the standard analysis of pupillometric data is often temporally coarse: averaging pupillary diameter across a trial or using the maximum dilation during a pre-defined temporal window for analysis, e.g., [12], [14]–[16]. Although means and maxima are useful in discriminating responses between categories (e.g., emotional vs. neutral sounds, [14]; effortful vs. easy mathematical calculations, [17]), these metrics ignore temporal information that could further elucidate the dynamics of attention to information.

**Figure 1:**
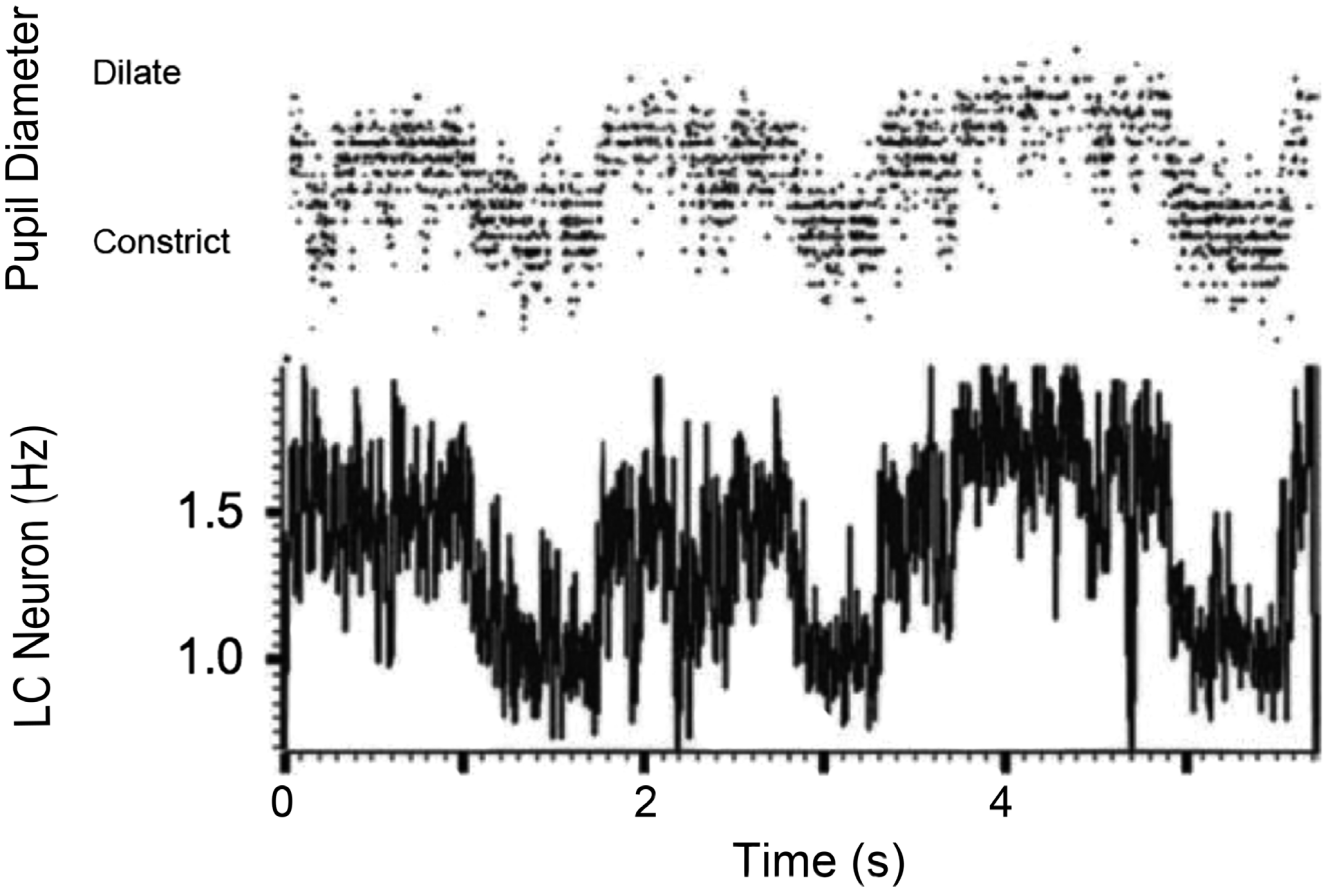
The relationship between pupil diameter and baseline locus coeruleus activity. This figure is adapted from Rajkowski, Kubiak, & Aston-Jones [19], and shows the close positive association between baseline locus coeruleus activity and tonic pupil diameter. Rajkowski and colleagues recorded from a single LC neuron in the monkey, and used a remote eye-tracking camera to measure pupil dilation during a target detection task.

There is ample reason to believe that the pattern of pupillary dilation dynamics, when recorded at high temporal resolution, can provide a continuous index of attention. Pupil dilations have been shown to closely track norepinephrine (NE) release by the locus coeruleus (LC) [18], [19] (see Figure 1). NE release in turn effects changes in individuals' attention and arousal by enhancing the synaptic responsivity of neurons to subsequent inputs [12]. As the LC is the sole source of NE-releasing fibers to the forebrain [18], and pupils can dilate with latencies as short as 200 ms [20], pupil size over time should provide an overt and objective measure of NE levels in the forebrain on a sub-second time-scale.

Previous studies have largely demonstrated the reliability of pupil diameter in acting as a reporter variable for LC activity in animal models, e.g. [19], [21], or have focused on the relationship between baseline rates of LC activity, tonic pupil diameter, and arousal states [19], [22]–[24]. Less focus has been placed on understanding what insight the pupillary time-course can offer when it comes to real-time human information processing. Recently, Smallwood and colleagues probed whether the temporal pattern of pupil dilation indexes information processing under conditions of attention [25]. Their paradigm differed from other studies investigating the relationship between pupil size and attentional state, e.g. [12], [26], in several key ways. First, their paradigm used the overall time-course of pupillary dilation as a dependent measure, rather than the peak dilation within a temporal window. Their design also allowed them to analyze only the time-periods during which participants viewed stimuli that did not require a response, avoiding potential confounds associated with motor planning and execution. In this way, they tested not only whether phasic dilations occurred during periods of high attention (which has been well-established in the literature: [27]–[29]), but also whether the specific timing of these dilations could be used to discriminate between conditions of task focus versus mind-wandering. The LC-NE system is associated with “alertness” for incoming external stimuli (exogenous attention [30], [31]), with sustained endogenous attention generally considered the responsibility of cholinergic pathways [30], [32], [33]. Thus, Smallwood and colleagues hypothesized that when participants were motivated to attend to exogenous cues, their pupil dilations would couple to the presentation of exogenous cues (“task-evoked dilations”) compared to when they were not motivated to attend to those cues [25]. Their results supported their hypothesis.

Consistent with their data, Smallwood and colleagues concluded that mind-wandering essentially “de-couples” participants from external stimuli. Further supporting this conclusion, recent research found that pupil dilations systematically varied with spontaneous fluctuations of engagement [34]. Neither study, however, makes strong claims as to *what about the stimuli* causes pupil dilation coupling under task focus, possibly because there are two viable explanations: (1) changes in low-level cues that are attended more in the task-focus condition (in Smallwood and colleagues' paradigm, these were luminance changes between fore- and background colors with the disappearance of the fixation cross and appearance of the next stimulus), and (2) changes in (high-level) information that necessarily accompanied each new stimulus. Here, we tested whether pupil dilations reflect attention *in the absence of luminance changes on the retina*. If so, this would rule out the first (luminance) explanation as the primary cause of Smallwood and colleagues' 2011 results, leaving the striking explanation that the pupil dilation time-course can index attention to high-level informational changes [25]. To do so, we directly replicated Smallwood and colleagues' paradigm while controlling for luminance changes.

## Materials and Methods

### Ethics Statement

This research was approved by Dartmouth College's Committee for the Protection of Human Subjects, Protocol #20951. Written informed consent was obtained from all participants at the start of the study.

### Participants

22 Dartmouth undergraduates (13 females) completed the same versions of both Choice Reaction Time (CR) and Working Memory (WM) tasks while eye-tracked, in exchange for course credit. Only individuals with normal vision, or vision corrected-to-normal using contact lenses, were allowed to participate in the study. Individuals wearing glasses were not used due to the propensity of some glasses to occlude the pupillary response. 16 participants (11 females) passed quality control cut-offs (see below); data from these participants is discussed below.

### Eye-tracking and Quality Control

Pupil diameter was collected from the left eye at 120 Hz using the ASL Eye-Trac 6 eye-tracker (Applied Sciences Laboratories, Bedford, MA). Any missing values (e.g., due to blinks or machine artifact) were interpolated using basic linear interpolation. In accordance with quality control measures instituted by Smallwood and colleagues [25], individuals whose data required in excess of 40% interpolation, and/or whose responses showed below-chance accuracy during experimental trials were rejected from analysis. Resultant pupil data was median filtered (order 5), low-pass filtered (cutoff frequency 10 Hz), and z-scored to account for individual differences in pupil size. In order to maintain any task-related differences in pupillary response, z-scoring was completed across both tasks per individual.

### Design and Procedure

**Figure 2:**
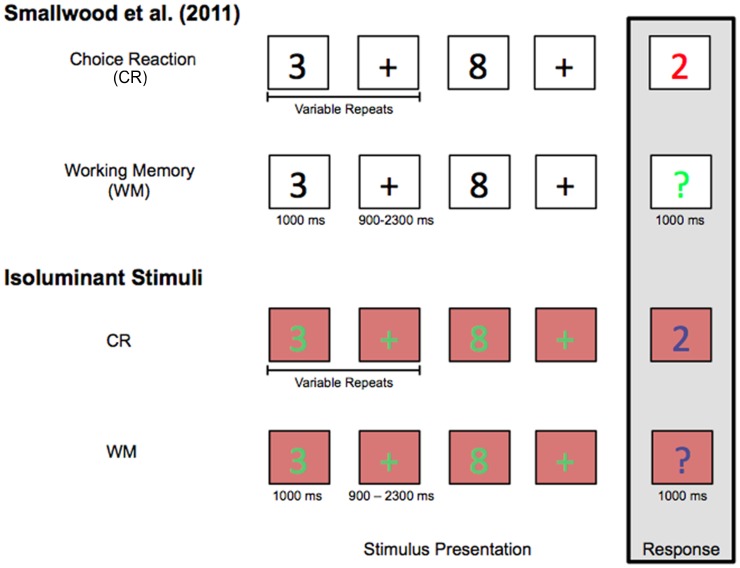
A schematic of experimental tasks used in Smallwood et al. [25] and the current paradigm. The current paradigm conducted an isoluminant replication of the design used in Smallwood et al. [25]. Participants completed both Choice Reaction and Working Memory tasks. In both conditions, they viewed and sporadically assessed the parity of numbers presented on the computer monitor. The Choice Reaction task only demanded attention to colored numbers; the Working Memory condition demanded attention to all presented numbers.

Design for CR and WM tasks replicated Smallwood et al. [25] (see Figure 2). In both tasks, strings of numbers interleaved with fixation crosses were shown to participants. Each number was presented for 1000 ms; fixation crosses appeared onscreen for between 900–2300 ms. Response trials occurred every 2–5 numbers, and were indicated by a colored probe. In the CR task, this response probe was a number, and participants were asked to indicate whether that number was even or odd. In the WM task, the probe was a colored question mark, and participants performed a 1-back task, indicating the parity of the *preceding* number. Importantly, the CR task did not require attention to non-probe trials, whereas the WM task demanded that participants attend to and remember each non-probe number. Each task was 10 minutes in duration, and contained 48 probes.

Smallwood and colleagues utilized response probes that were colored either red or green, on a white background. Non-probe numbers were black. Given pupillary sensitivity to luminance [35], the current experiment used an isoluminant paradigm. This allowed us to test whether the pupils dynamically coupled to attended stimuli in the absence of luminance changes. To determine the RGB values perceived as isoluminant by each individual, participants first completed a short color task, coded in Matlab and presented on a Cathode Ray Tube (CRT) monitor. A CRT monitor was used because of its more sensitive refresh rate. In this task, participants saw a small box colored red, green, or blue on a gray background. For any value that was not isoluminant with the gray background, this box would appear to flicker. Participants used the keyboard arrow keys to adjust the luminance of these boxes, and save the values at which flickering ceased for each color (i.e., at which isoluminance was reached). The corresponding RGB values were then used to tailor participant-specific CR and WM tasks, presented using E-Prime 2.0 Professional software on the CRT monitor. In this paradigm, participants viewed strings of green non-probes and fixation crosses on a red background, and responded when blue probes appeared (see Figure 2). To control for eye movements, participants were instructed to keep their gaze on the fixation cross and stimuli that appeared in the center of the computer screen throughout the duration of the experiment. Onscreen luminance remained constant, with only stimulus salience differing across tasks. The order in which these tasks were completed was counterbalanced across subjects. Participants sat approximately 30 inches from the eye-tracker. A chin rest was utilized in order to keep participants stationary during the task.

## Results

**Figure 3:**
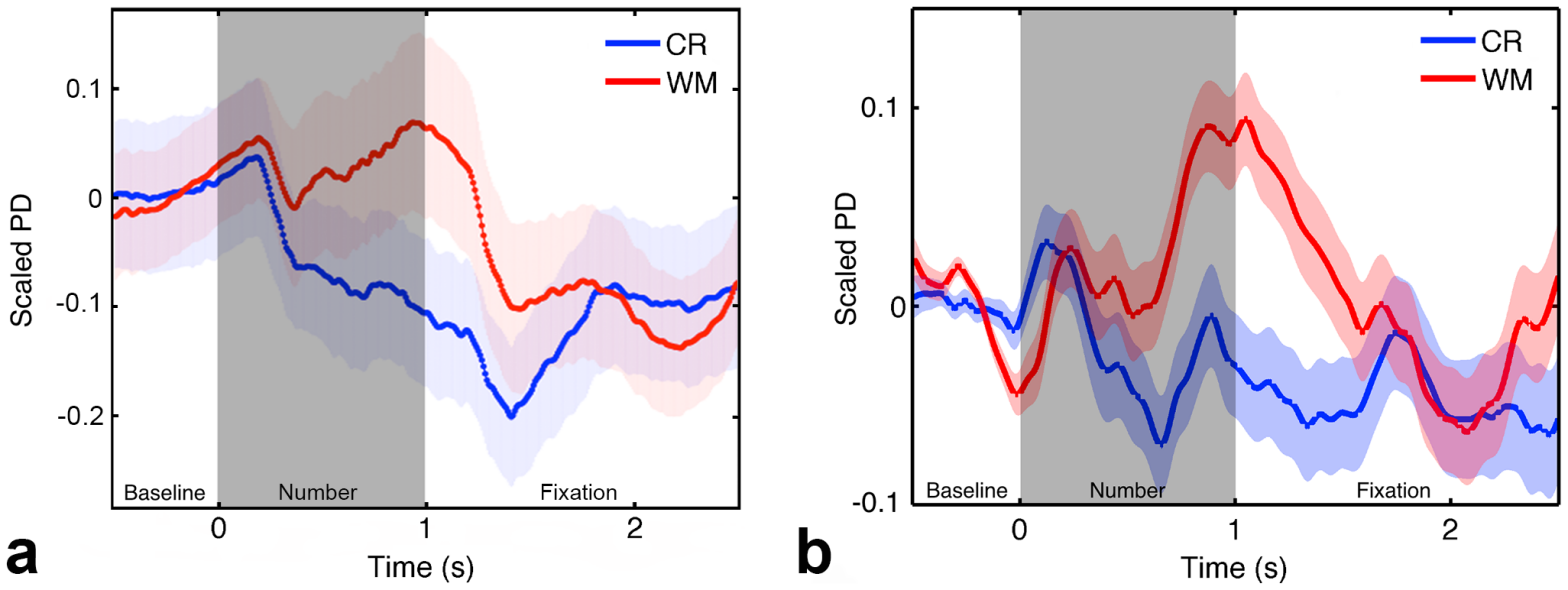
Pupil dilation patterns in response to information differ as a function of attention. (A) Pupils exhibited task-evoked dilations to stimuli only when they were task-relevant, *F*(2.841, 42.621) = 5.818, *p*<.002. Error bars indicate one standard error of the mean. Given controlled luminance across conditions, we attribute this coupling to the information carried by the stimulus, and not to changes in low-level luminance. (B) Smallwood and colleagues' findings (adapted from [25]) for comparison.

Following the procedure outlined in Smallwood et al. [25], analyses were constrained to participants' pupillary behavior in response to presentation of non-probes, as only these differed in salience across conditions. Pupillary time-series data were locked to the 2.5 seconds following presentation of each of the 169 non-probes for each participant, for each task. These time-courses were normalized across both tasks to preserve their responses across CR and WM conditions. Time-courses were then averaged into 10 250 ms bins (with the first bin encompassing [0–250 ms]), and analyzed using a 2 (Task)×10 (Time) Repeated Measures ANOVA. There was no main effect of Task, but there was a main effect of Time (*F*(2.422, 36.325) = 6.165, *p*<.003, *d* = .641, power = .905) irrespective of task. Supporting Smallwood and colleagues' findings, there was a significant Task×Time interaction (*F*(2.841, 42.621) = 5.818, *p*<.002, *d* = .622, power = .923) indicating that there were differences in the pupil response to non-probe stimuli between the two tasks (see Figures 3a and 3b). Contrasts revealed that pupil dilations were significantly larger in the WM condition than in the CR condition in three time periods (bin 5 [vs. bin 4, *F*(1,1) = 4.871, *p* = .043], bin 6 [vs. bin 7, *F*(1,1) = 7.717, *p* = .014], and bin 7 [vs. bin 8, *F*(1,1) = 9.573, *p* = .007]) after stimulus onset (see Figure 3a). These bins corresponded to the time period occurring 1–1.75 seconds following onset of the non-probe stimulus. This is consistent with findings that pupillary dilations in response to cognitive tasks peak approximately 1 second after stimulus onset [36], and suggest that task-evoked responses were present only in the WM condition. There was no significant difference in the number of blinks participants made (*t*(15) = −.278, *p* = *ns*) or their percent accuracy (*t*(15) = −.901, *p* = *ns*) across WM and CR tasks. We also found no significant difference in tonic pupil size prior to probes as a function of accuracy (*t*(15) = −.126, *p* = *ns*) within task.

**Figure 4:**
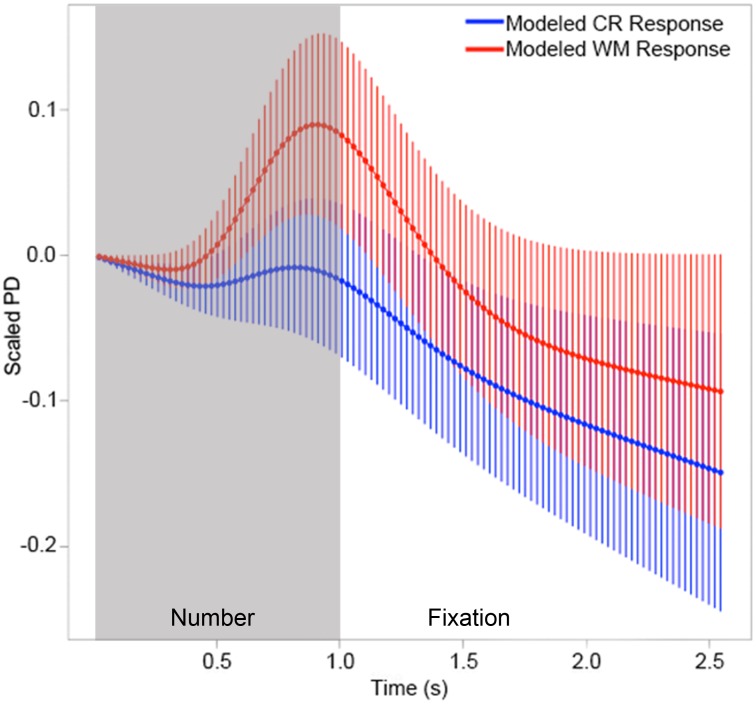
Attentional pulse strengths of non-probe stimuli differ as a function of attention. Wierda and colleagues' automated pupil dilation deconvolution algorithm [36] was used to derive the attentional pulse strengths associated with non-probe stimuli in WM and CR conditions. This attentional pulse was significantly stronger in the WM condition (*M1* = .254, *SD1* = .28) than in the CR condition (*M2* = .064, *SD2* = .11), *t*(15) = 2.333, *p* = .03. The deconvolved pupillary responses to non-probes are displayed above, with error bars indicating one standard error of the mean.

We also deconvolved the pupillary responses associated with each condition, using the automated pupil dilation deconvolution method recently developed by Wierda and colleagues [36]. Each participant's normalized mean response for the 2.5 seconds following stimulus onset was downsampled to 40 Hz, and averaged into 99 25 ms bins. The algorithm deconvolved these responses by calculating the misfit between the observed and predicted pupil dilation patterns, based on the pupillary response function of Hoeks and Levelt [37], and calculated the average strength of the attentional pulse associated with both conditions. We compared these attentional strengths using a paired t-test. The attentional pulse associated with non-probes in the WM condition (*M1* = .254, *SD1* = .28) was significantly stronger than the pulse associated with CR non-probes (*M2* = .064, *SD2* = .11), *t*(15) = 2.333, *p* = .03. The deconvolved pupillary responses associated with WM and CR conditions are shown in Figure 4.

## Discussion

Five decades of pupillometric research left the tantalizing impression that pupillary dynamics offer a real-time window on attention. Recently, Smallwood and colleagues [25] came closest to achieving this possibility. Here, we extend their results by controlling for low-level (luminance) changes. We replicated Smallwood and colleagues' finding that the dynamic pupillary response discriminates trials during which participants are attending versus not attending exogenous cues. That is, pupillary dilations were time-locked to the appearance of exogenous cues only when those cues were attended, and this effect did not depend on low-level luminance changes accompanying those cues.

The current findings suggest that the dynamics of pupil dilation comprise a high-temporal resolution measure of attention. Here, we have shown that pupil dilations are capable of indexing information changes independent of low-level visual changes (luminance). The objectivity of pupillometry and the relative ease with which it can be obtained, renders it an ideal metric for a multitude of research questions. Although the Smallwood et al. paradigm [25] uses pupil dilation dynamics to track the changing salience of discrete visual stimuli, we see no reason why these pupillary dynamics could not be used to index moment-by-moment fluctuations of attention to other input modalities (e.g., audition and touch). While the concept of using pupil dilation dynamics as an index of cognitive processing is not novel [17], it remains underutilized. Recent technological advances now make it possible to achieve the temporal resolution necessary to measure real-time fluctuations in attention – not just to changes in light but to changes in information. Pupil dilation dynamics thus afford a temporally-sensitive portal onto the processing of another mind.

## References

[1] Looser CE, Wheatley TP (2010) The tipping point of animacy: How, when, and where we perceive life in a face. Psychol Sci 21: 1854–1862. 21097720 10.1177/0956797610388044

[2] Whalen PJ, Kagan J, Cook RG, Davis FC, Kim H, et al (2004) Human amygdala responsivity to masked fearful eye whites. Sci 306: 2601. 10.1126/science.1103617 15604401

[3] Siegle GJ, Ichikawa N, Steinhauer S (2008) Blink before and after you think: Blinks occur prior to and following cognitive load indexed by pupillary responses. Psychophysiol 45: 679–687. 10.1111/j.1469-8986.2008.00681.x 18665867

[4] Langton SRH, Watt RJ, Bruce V (2000) Do the eyes have it? Cues to the direction of social attention. Trends Cogn Sci 4: 50–59. 10652522 10.1016/s1364-6613(99)01436-9

[5] Bradley MM, Miccoli L, Escrig MA, Lang PJ (2008) The pupil as a measure of emotional arousal and autonomic activation. Psychophysiol 45: 602–607. 10.1111/j.1469-8986.2008.00654.x PMC3612940 18282202

[6] Chapman CR, Oka S, Bradshaw DH, Jacobson RC, Donaldson GW (2003) Phasic pupil dilation response to noxious stimulation in normal volunteers: Relationship to brain evoked potentials and pain report. Psychophysiol 36: 44–52. 10.1017/s0048577299970373 10098379

[7] Kahneman D, Beatty J (1966) Pupil diameter and load on memory. Sci 154: 1583–1585. 10.1126/science.154.3756.1583 5924930

[8] Beatty J, Lucero-Wagoner B (2000) The pupillary system. In: Cacioppo JT, Tassinary LG, Berntson GG, editors. Handbook of psychophysiology. Hillsdale, NJ: Cambridge University Press. pp. 142–162.

[9] Goldwater BC (1972) Psychological significance of pupillary movements. Psychol Bull 77: 340–355. 5021049 10.1037/h0032456

[10] Heller PH, Perry F, Jewett DL, Levine JD (1990) Autonomic components of the human pupillary light reflex. Invest Ophthalmol Vis Sci 31: 156–162. 2137115

[11] MacLachlan C, Howland HC (2002) Normal values and standard deviations for pupil diameter and interpupillary distance in subjects aged 1 month to 19 years. Opthalmic Physiol Opt 22: 175–182. 10.1046/j.1475-1313.2002.00023.x 12090630

[12] Gilzenrat MS, Nieuwenhuis S, Jepma M, Cohen JD (2010) Pupil diameter tracks changes in control state predicted by the adaptive gain theory of locus coeruleus function. Cogn Aff Behav Neurosci 10: 252–269. 10.3758/CABN.10.2.252 PMC3403821 20498349

[13] Hess EH, Polt JM (1960) Pupil size as related to interest value of visual stimuli. Sci 132: 349–350. 10.1126/science.132.3423.349 14401489

[14] Partala T, Surakka V (2003) Pupil size variation as an indication of affective processing. Hum Comp Stud 59: 185–198.

[15] Ariel R, Castel AD (2014) Eyes wide open: Enhanced pupil dilation when selectively studying important information. Exp Brain Res 232: 337–344. 24162863 10.1007/s00221-013-3744-5 PMC3895405

[16] Bradley MM, Miccoli L, Escrig MA, Lang PJ (2008) The pupil as a measure of emotional arousal and autonomic activation. Psychophysiol 45: 602–607. 10.1111/j.1469-8986.2008.00654.x PMC3612940 18282202

[17] Hess EH, Polt JM (1964) Pupil size in relation to mental activity during simple problem-solving. Sci 143: 1190–1192. 10.1126/science.143.3611.1190 17833905

[18] Berridge CW, Waterhouse BD (2003) The locus coeruleus-noradrenergic system: Modulation of behavioral state and state-dependent cognitive processes. Brain Res Rev 42: 33–84. 12668290 10.1016/s0165-0173(03)00143-7

[19] Rajkowski J, Kubiak P, Aston-Jones G (1993) Correlations between locus coeruleus (LC) neural activity, pupil diameter, and behavior in monkey support a role of LC in attention. Soc Neurosci Abs 19: 974.

[20] Lowenstein O, Loewenfeld IE (1962) The pupil. In: H Davidson, editor. The eye. New York: Academic Press. pp. 255–340.

[21] Rajkowski J, Majczynski H, Clayton E, Aston-Jones G (2004) Activation of monkey locus coeruleus neurons varies with difficulty and performance in a target detection task. J Neurophysiol 92: 361–371. 15028743 10.1152/jn.00673.2003

[22] Hou RH, Freeman C, Langley RW, Szabadi E, Bradshaw CM (2005) Does modafinil activate the locus coeruleus in man? Comparison of modafinil and clonidine on arousal and autonomic functions in human volunteers. Psychopharmacol 181: 537–549. 10.1007/s00213-005-0013-8 15983798

[23] Morad Y, Lemberg H, Yofe N, Dagan Y (2000) Pupillography as an objective indicator of fatigue. Curr Eye Res 21: 535–542. 11035533

[24] Phillips MA, Szabadi E, Bradshaw CM (2000) Comparison of the effects of clonidine and yohimbine on spontaneous pupillary fluctuations in healthy human volunteers. Psychopharmacol 150: 85–89. 10.1007/s002130000398 10867980

[25] Smallwood J, Brown KS, Tipper C, Giesbrecht B, Franklin MS, et al (2011) Pupillometric evidence for the decoupling of attention from perceptual input during offline thought. PLoS One 6: e18298. 21464969 10.1371/journal.pone.0018298 PMC3064669

[26] Gabay S, Pertzov Y, Henik A (2011) Orienting of attention, pupil size, and the norepinephrine system. Atten Percept Psychophys 73: 123–129. 21258914 10.3758/s13414-010-0015-4

[27] Beatty J (1982) Task-evoked pupillary responses, processing load, and the structure of processing resources. Psychol Bull 91: 276–292. 7071262

[28] Janisse MP (1973) Pupil size and affect: A critical review of the literature since 1960. Can Psychol 14: 311–329.

[29] Privitera CM, Renninger LW, Carney T, Klein S, Aguilar M (2008) The pupil dilation response to visual detection. In: Rogowitz BE, Pappas TN, editors. Proceedings of the SPIE-IS&T electronic imaging. San Jose, CA: SPIE. pp. 68060T-68060T-11.

[30] Colombo J (2001) The development of visual attention in infancy. Annu Rev Psychol 52: 337–367. 11148309 10.1146/annurev.psych.52.1.337

[31] Tse PU (2013) The neural basis of free will: Criterial causation. Cambridge, MA: MIT Press.

[32] Himmelheber AM, Sarter M, Bruno JP (2000) Increases in cortical acetylcholine release during sustained attention performance in rats. Cogn Brain Res 9: 313–325. 10.1016/s0926-6410(00)00012-4 10808142

[33] Muir JL, Everitt BJ, Robbins TW (1994) AMPA-induced excitotoxic lesions of the basal forebrain: A significant role for the cortical cholinergic system in attentional function. J Neurosci 14: 2313–2326. 7512637 10.1523/JNEUROSCI.14-04-02313.1994 PMC6577145

[34] Franklin MS, Broadway JM, Mrazek MD, Smallwood J (2013) Schooler, JW (2013) Window to the wandering mind: Pupillometry of spontaneous thought while reading. Q J Exp Psychol 66: 2289–2294. 10.1080/17470218.2013.858170 24313285

[35] De Groot SG, Gebhard JW (1952) Pupil size as determined by adapting luminance. J Opt Soc Am 42: 492–295. 14939111 10.1364/josa.42.000492

[36] Wierda SM, van Rijn H, Taatgen NA, Martens S (2012) Pupil dilation deconvolution reveals the dynamics of attention at high temporal resolution. Proc Natl Acad Sci U S A 109: 8456–8460. 22586101 10.1073/pnas.1201858109 PMC3365158

[37] Hoeks B, Levelt WJM (1993) Pupillary dilation as a measure of attention: A quantitative system analysis. Behav Res Meth Instrum Comput 25: 16–26.

